# Cancer risk is not increased after conventional hip arthroplasty: A nationwide study from the Finnish Arthroplasty Register with follow-up of 24,636 patients for a mean of 13 years

**DOI:** 10.3109/17453671003667150

**Published:** 2010-03-31

**Authors:** Tuomo Visuri, Pekka Pulkkinen, Pekka Paavolainen, Eero Pukkala

**Affiliations:** ^1^Research Department, Centre for Military Medicine; ^2^Department of Public Health, University of Helsinki; ^3^Hospital Orton; ^4^Finnish Cancer Registry, Helsinki and School of Public Health, University of TampereFinland

## Abstract

**Background and purpose:**

Wear debris from conventional total hip arthroplasty (THA) induces chromosomal aberrations and DNA damage, which may promote cancerogenesis. A long latent period is required for solid tumors. We therefore re-analyzed a large THA cohort for cancer.

**Patients and methods:**

We updated a cohort of 24,636 patients with primary osteoarthritis and metal-on-polyethylene THA who had been entered in the Finnish Arthroplasty Register between 1980 and 1995, and linked it to the Finnish Cancer Registry for cancer risk assessment up to 2005. The mean follow-up time was 13 years. The numbers of cancer cases observed were compared with expected rates based on incidence in the general population.

**Results:**

The standardized incidence ratio (SIR) for the whole follow-up period was 0.95 (95% confidence interval (CI): 0.92–0.97). After 10 years of follow-up, the SIR was equal to that in the normal population (SIR = 0.98, 95% CI: 0.94–1.03). Incidence of lung cancer was low throughout the follow-up time and that of prostate cancer was slightly elevated. The incidence rates for all other forms of cancer did not deviate significantly from those in the normal population.

**Interpretation:**

We found no increased cancer risk in patients with conventional THA after an average of 13 years and up to 25 years of follow-up.

## Introduction

Corrosion and wear of THA liberates metal, polyethylene, and methacrylate particles. According to post-mortem and in vivo studies, such metal particles are widely dispersed in the body and have been found in the liver, the spleen, and in local and distant lymph nodes (Langkamer et al. 1992, Case et al. 1994, Eng et al. 1997, Shea et al. 1997, Urban et al. 2000, 2004). Thus, patients with metal-on-polyethylene (MP) bearings are permanently exposed to Cr, Co, and Ti ions more extensively than healthy controls (Jacobs et al. 1998, Savarino et al. 2002, MacDonald et al. 2003, Rasquinha et al. 2006).

Both in vivo and in vitro studies have shown chromosomal aberrations and DNA damage in association with debris from CoCr and TiAlV prostheses. Bone marrow adjacent to the worn implant was found to show an increased number of chromatid breaks and gaps (Case et al. 1996). At revision THA, patients with mixed types of CoCr prostheses had a 2.5-fold increase in peripheral blood lymphocytes with aneuploidy and 3.5 fold more chromosomal translocations than at primary THA (Doherty et al. 2001). Wear debris from worn hip and knee prostheses in human tissue cultures has been found to damage chromosomes in a dose-dependent manner, which was specific to the type of metal. A correlation was found between Co and Cr concentrations on the one hand and chromosomal breakage and aneuploidy on the other; similarly, there was a correlation between Ti concentrations and aneuploidy (Daley et al. 2004). Davies et al. (2005) showed that synovial fluid from revised CoCr metal-on-metal (MM) and MP prostheses caused DNA damage to human fibroblast culture cells, which was believed to be a synergistic effect of Co and Cr ions. Wear debris from a worn TiAlV MP prosthesis has been found to produce chromosomal instability in human fibroblast cell cultures (Coen et al. 2003). Further genomic instability was evident when the progeny of the cells were exposed to low doses of wear debris. Chromosomal aberrations associated with metallic ions can be predictive of cancerogenesis (Bonassi et al. 2000) and an increased risk of cancer in THA patients. Latent periods of 10 or more years are required for the development of particle-, virus-, or industrial metal-induced cancers (zur Hausen 1996, Yates et al. 1997, Anttila et al. 1998). We investigated the long-term risks for all and site-specific cancers in primary THA patients with MP prostheses by updating our previous cohort study from the Finnish Endoprosthesis Register (Paavolainen et al. 2000).

## Patients and methods

The data from the Finnish Arthroplasty Register was linked with that from the Finnish Cancer Registry, and records of all cancer cases were obtained from 1980 through 2005. The original cohort comprised 33,694 patients, 24,636 of whom were operated on for primary osteoarthritis (OA). All patients were provided with MP prostheses, and 71% of the femoral components and 65% of the acetabular components were cemented. The stem material was cobalt chrome molybdenum (CoCrMo) in about 40% of the prostheses, titanium aluminium vanadium (TiAlV) in 36%, and stainless steel (SS) in 24% (Nevalainen et al. 2000).

The patients were followed up for cancer incidence from the date of first operation until death, or until 31 December 2005. The follow-up time was up to 25 years with a mean of 12.6 years. The follow-up was not stopped at the date of first diagnosis of cancer, but all primary cancers diagnosed during the follow-up period were counted as observed cases. The numbers of observed cases for each cancer and person-years at follow-up were stratified by sex, calendar period and 5-year age group, and follow-up time since the operation. The expected number of each cancer type was calculated by applying the number of person-years in each stratum to the corresponding cancer incidence rate in the Finnish population. The calendar periods used were 1980–1986, 1987–1993, 1994–1999, and 2000–2005, and the follow-up categories were 0–2, 3–9, 10–20, and > 20 years since the operation. Only 97 patients (0.04%) were followed for more than 20 years. The relative risk of cancer was expressed as the ratio of observed and expected number of cases, i.e. standardized incidence ratio (SIR). The 95% confidence intervals (95% CIs) were defined assuming that the number of observed cases followed a Poisson distribution.

## Results

The cohort yielded 310,341 person-years, which is approximately twice as much as in the previous analysis (Paavolainen et al. 2000). The proportion of male patients was 38% (Table 1). Altogether, 13,324 patients (54%) had died before the end of 2005. There were no losses to follow-up.

**Table 1.** Numbers of patients (n) by age at operation, and number of person-years at end of follow-up

Age	Men		Women	
	n	Person-years	n	Person-years
15–29	10	35	11	44
30–44	156	784	168	906
45–59	2,060	11,144	2,214	11,796
60–74	5,751	57,484	9,214	83,132
≥ 75	1,502	43,861	3,550	101,155
Total	9,479	113,308	15,157	197,033

The overall SIR for all-site cancers was 0.95 (95% CI: 0.92–0.97) (Table 2). After the first 2 postoperative years with very low all-site cancer incidence (SIR = 0.80; 95% CI: 0.73–0.87), the SIR rose to 0.97 (95% CI: 0.94–1.00) (Table 3).

**Table 2.** Observed (Obs) numbers of cancer cases and standardized incidence ratios (SIRs) with 95% confidence intervals, by site

Primary site	Obs	SIR	95% CI
All sites	4846	0.95	0.92–0.97 **^a^**
Lip	50	1.19	0.88–1.56
Mouth, pharynx	104	1.09	0.89–1.30
Esophagus	49	0.81	0.60–1.06
Stomach	245	0.95	0.84–1.07
Colon	310	0.89	0.79–0.99 **^b^**
Rectum	193	0.91	0.79–1.04
Liver	65	0.87	0.67–1.11
Gall bladder	86	1.01	0.81–1.24
Pancreas	201	0.90	0.78–1.02
Larynx	19	0.80	0.48–1.25
Lung	368	0.65	0.58–0.71 **^a^**
Breast	515	1.00	0.92–1.08
Uterus, cervix	28	1.03	0.68–1.48
Uterus, corpus	134	0.94	0.79–1.10
Ovary	86	0.94	0.75–1.16
Prostate	843	1.09	1.02–1.16 **^b^**
Kidney	180	1.08	0.93–1.24
Bladder,ureter, urethra	222	0.98	0.86–1.11
Melanoma	97	0.99	0.80–1.20
Skin	279	1.07	0.95–1.20
Brain/nervous system	103	0.97	0.79–1.16
Thyroid	38	1.06	0.75–1.45
Bone	3	0.72	0.15–2.10
Connective tissue	24	0.90	0.58–1.34
Non-Hodgkin's lymphoma	167	0.92	0.79–1.06
Nodal non-Hodgkin's lymphoma	98	0.81	0.66–0.98 **^b^**
Hodgkin's lymphoma	13	1.26	0.67–2.15
Myeloma	90	1.14	0.92–1.40
Leukemia	96	0.85	0.69–1.03
**^a^** p < 0.001; **^b^** p < 0.05.			

**Table 3.** Observed (Obs) numbers of cancer cases and standardized incidence ratios (SIR) with 95% confidence intervals (CI), by site and the time since the first total hip arthroplasty

Primary site				Time since operation								
	0–2 years			3–10 years			11–20 years			> 20 years		
	Obs	SIR	95% CI	Obs	SIR	95% CI	Obs	SIR	95% CI	Obs	SIR	95% CI
All sites	492	0.80	0.73–0.87 **^a^**	2560	0.95	0.92–0.98 **^b^**	1697	0.99	0.94–1.03	97	0.93	0.75–1.12
Stomach	31	0.82	0.55–1.15	134	0.95	0.80–1.11	79	1.07	0.85–1.33	1	0.24	0.01–1.34
Colon	30	0.79	0.53–1.12	165	0.92	0.78–1.06	107	0.87	0.72–1.04	8	1.01	0.44–1.98
Rectum	19	0.72	0.43–1.12	111	0.98	0.81–1.17	59	0.86	0.66–1.11	4	0.98	0.27–2.51
Pancreas	21	0.78	0.48–1.18	111	0.95	0.78–1.13	62	0.82	0.63–1.05	7	1.41	0.57–2.90
Lung, trachea	40	0.48	0.34–0.65 **^a^**	204	0.66	0.57–0.75 **^a^**	115	0.70	0.57–0.82 **^a^**	9	1.00	0.46–1.89
Breast	69	1.02	0.79–1.29	264	0.96	0.85–1.08	177	1.08	0.93–1.24	5	0.49	0.16–1.14
Female genital												
organs	40	0.97	0.69–1.32	152	0.95	0.80–1.10	82	0.88	0.70–1.09	5	0.88	0.29–2.05
Kidney	20	0.91	0.56–1.41	100	1.11	0.90–1.34	55	1.05	0.79–1.36	5	1.65	0.53–3.84
Prostate	56	0.81	0.61–1.05	433	1.10	1.00–1.20	335	1.14	1.02–1.26 **^b^**	19	1.16	0.70–1.80
Melanoma	10	0.88	0.42–1.61	50	0.99	0.74–1.30	33	0.97	0.67–1.36	4	1.84	0.50–4.71
>Lymphoma,												
leukemia	41	0.68	0.49–0.92	265	1.00	0.88–1.13	153	1.11	0.94–1.30	2	0.19	0.20–0.70 **^b^**
**^a^** p < 0.001; **^b^** p < 0.05.												

After 10 and 20 years of follow-up, the all-site cancer incidence was similar to that in the general population with a SIR of 0.98 (95% CI: 0.94–1.03) and 0.93 (95% CI: 0.75–1.12), respectively. Female patients had higher SIR values than male patients during the first 2 years (0.91 (95% CI: 0.81–1.01) vs. 0.69 (95% CI: 0.60–0.78)), and between years 3 and 9 postoperatively (0.98 (95% CI: 0.92–1.02) vs. 0.93 (95% CI: 0.88–0.98)). These higher SIR values were mainly due to breast cancer and female genital cancers. The corresponding SIR values in the general population were, however, similar from the beginning and throughout the whole study period (Table 3). Site-specific cancers, except for lung, colon, prostate cancer and non-Hodgkin's lymphoma, had SIR values that were similar to those in the normal population (Table 2). Lung cancer incidence was low in the male patients throughout the whole follow-up period (with SIR values of 0.35–0.84) (Table 3).

SIR for kidney cancer was relatively high already during the first postoperative year and increased slightly thereafter (Tables 2 and 3). A slight excess of prostate cancers was observed between years 10 and 19 (SIR = 1.14) and also during the whole follow-up (SIR = 1.09) (Table 3). The SIR for bone and soft tissue sarcomas was 0.77 (95% CI: 0.51–1.12).

A slight increase in hematopoetic cancers (SIR = 1.11) was observed between years 10 and 19 postoperatively, which was due to an increase in multiple myelomas and Hodgkin lymphomas. SIRs for all hematopoetic cancers were similar to those in the normal population (0.97). The SIRs for non-Hodgkin's lymphomas and leukemia remained below (1.00) during the whole follow-up time, whereas those for multiple myeloma ranged from 1.00 to 1.19, and those for Hodgkin's lymphoma from 1.13 to 1.38 after the twentieth postoperative year.

## Discussion

The patients in our cohort were exposed to the degradation products of the most common metal alloys used: CoCrMo, TiAlV, or SS alloy, but cement usage and different metal compositions and cancer risk were not studied because these data were not available per hip or per patient in the study material.

The overall cancer risk seen in this study (SIR = 0.95) was the same level (SIR = 0.98) to that seen in a large meta-analysis on THA patients with 1,094 000 person-years and operated on for all indications, but with a mean follow-up time of only 6 years (Onega et al. 2006). The same findings were reported by Goldachre et al. (2005) from the UK after an average of 8 years of follow-up of 33,691 patients who were operated for all indications. The SIR of 0.92 in our cohort during the first 10 years is equal to that (0.93) observed in a Nordic meta-analysis in patients who were operated on for OA with 374,000 person-years and with a mean follow-up time of 8 years (Visuri et al. 2003). In one study, OA has been reported to be associated with low cancer co-morbidity: Thomas et al. (2000) reported a low all-site cancer incidence (0.85 for males and 0.83 for females) in patients with all forms of OA in a Scottish cohort.

Pre-screening of our patients for surgery decreased the cancer incidence in a comparison with the normal population approximately for 10 years indicating a long latent period. We found 20% less cancer cases than in a comparative sample of the general Finnish population during the 2 postoperative years, but after 11 years of follow-up the cancer incidence rate were equalized.

Lung cancer incidence in the male patients was low throughout the whole follow-up period. It is well known that tobacco smoking is the cause of lung cancer in 90% of cases (Tyczynsky et al. 2003). A low incidence of lung cancer was reported in a meta-analysis involving THA patients operated on for primary osteoarthritis (SIR = 0.69) (Visuri et al. 2003). In THA patients operated on for all indications, the SIR for lung cancer was found to be 0.82 (Onega et al. 2006), which was similar to the value from a large British cohort (0.86) (Goldachre et al. 2005). In 1,495 male Swedish constructors who smoked, the risk of severe osteoarthritis of the hip requiring surgery was found to be lower than in non-smokers or ex-smokers (Järvholm et al. 2005). Also, a significantly lower risk of hip osteoarthritis was found in 210 men who were current cigarette smokers than in men who had never smoked and who were listed for THA (Cooper et al. 1998). This association was not observed in women. Vingård et al. (1997) observed a reversed risk in 230 women who had undergone a THA operation.

Special interest lay in the occurrence of hematopoetic malignancies, given the high number of these cancers reported in earlier studies (Gillespie et al. 1988, Visuri and Koskenvuo 1991). The reticuloendothelial system is especially loaded with wear debris (Langkamer et al. 1992, Case et al.1996). In vivo studies on lymphocytes in THA patients, both patients with TiAlV and with CoCr prostheses, showed that they had chromosomal aberrations (Stea et al. 2000, Doherty et al. 2001). In addition, patients with MP bearings had a reduced number of peripheral T lymphocytes (Granchi et al. 2003). In our study, lymphomas and leukemias showed slightly increased SIR values until 20 years of follow-up, and after that a decrease to 0.19—but with only 2 cases observed and 10.5 cases expected. The incidence of sarcoma was low, and to our knowledge, our series did not include any tumor at the site of the prosthesis. Despite the vast number of implanted THAs, only 52 malignant tumors located close to an arthroplasty (46 sarcomas, 5 lymphomas, and 1 epidermoid carcinoma) have been reported in the western literature (Lucas et al. 2001, Schuh et al. 2004, Visuri et al. 2006, Hsieh et al. 2007, Min et al. 2008).

Until now, meta-analyses on the risk of cancer after THA have shown that the patients can effectively resist the genotoxic effects of Co and Cr particles and ions and the possible development of cancer (Visuri et al. 2003, Onega et. al 2006). Co and Cr are essential trace elements, and one can assume that the cells have inherited efficient repair systems for DNA damage caused by these elements (Reynolds et al. 2004). In addition, macrophages have been shown to counteract the genotoxic and cytotoxic effects of CoCr particles in vitro (Papageorgiou et al. 2008).

Our study with a mean follow-up time of 12.6 years did not show any all-site or site-specific risk of cancer in patients with conventional THA, and most THA patients will outlive any possible latent period for cancer. However, a 20–40-year latent period is known to be required for some solid malignant tumors (zur Hausen 1996, Yates et al. 1997, Anttila et al. 1998). As this survey may have been too short to ascertain the risk of cancer in young patients expected to use their THA prostheses for more than 30 years, analyses with even longer follow-up times are still needed.
